# Ultrasonographic (TCS) and clinical findings in overlapping phenotype of essential tremor and Parkinson’s disease (ET-PD)

**DOI:** 10.1186/1471-2377-14-54

**Published:** 2014-03-22

**Authors:** Kristina Laučkaitė, Daiva Rastenytė, Danguolė Šurkienė, Birutė Vaidelytė, Gabrielė Dambrauskaitė, Andrius Sakalauskas, Antanas Vaitkus, Rymantė Gleiznienė

**Affiliations:** 1 Department of Neurology, Lithuanian University of Health Sciences, Academy of Medicine, Mickevičiaus street 9, Kaunas LT-44307, Lithuania; 2 Kaunas University of Technology, Biomedical Engineering Institute, Studentų street Kaunas, Lithuania; 3 Department of Radiology, Lithuanian University of Health Sciences, Academy of Medicine, Mickevičiaus street 9, Kaunas, Lithuania

**Keywords:** Essential tremor, Parkinson’s disease, Hyperechogenicity of the substantia nigra, Phenotype, Transcranial sonography

## Abstract

**Background:**

Essential tremor (ET) and Parkinson’s disease (PD) are considered distinct disorders. The aim of the study was to look for a link or any distinguishing features by transcranial sonography (TCS), together with the clinical examination findings in a group of patients with overlapping phenotype of ET and PD (ET-PD).

**Methods:**

A prospective observational case-control study was carried out from the 3^rd^ January 2011 until 30^th^ January 2013 at the Hospital of Lithuanian University of Health Sciences. The final study group consisted of 15 patients with ET-PD, 116 patients with ET-only and 141 patients with PD-only. The control group included 101 subjects. Clinical diagnosis was of a diagnostic standard.

**Results:**

The main ultrasonographic findings in the ET-PD group were similar to those of the PD-only: hyperechogenicity of the substantia nigra (66.7%, p < 0.001) and nuclei raphe interruptions/absence (38.5%, p < 0.001). The single distinguishing TCS finding in ET-PD group was a lentiform nucleus hyperechogenicity (26.7%), however this was only significant when compared to controls (p = 0.006). An asymmetrical onset of symptoms (73.3%) in ET-PD group was characteristic to PD-only. The ET-PD patients had the longest disease duration (median 6 years, p < 0.001), the most frequent rate of positive family history (53.3%, p = 0.005), rather low prevalence of cogwheel rigidity (26.7%, p < 0.001), and higher mean Hoehn & Yahr scores compared to PD-only (2.6 ± 0.8 vs. 1.8 ± 0.8, p = 0.012).

**Conclusions:**

The main TCS findings of the present study in patients with overlapping ET-PD phenotype were similar to the PD-only group. The highest positive family history rate among ET-PD patients indicates a strong hereditary predisposition and needs genetic underpinnings. Some ET patients, who look like they may be developing co-morbid PD clinically, may have an alternative diagnosis for Parkinsonism, which could be delineated by TCS examination.

## Background

Essential tremor (ET) is one of the most common movement disorders, followed by Parkinson’s disease (PD) [1,2]. ET affects up to 14% and PD up to 1% of the population aged 65 years and over [3,4]. ET and PD are considered distinct disorders. However, an overlap of some clinical features in individual patients may appear [5]. Since the diagnosis of both ET and PD remains based only on the clinical criteria, many questions regarding a nosological association remain unanswered [4-11]. Misdiagnosis between the two is still common due to a lack of objective differential measurement [12].

Another debated controversy is whether ET should be viewed as a mono-symptomatic (“benign”) disorder, or whether it is a syndrome of several diseases including neurodegenerative [6,13,14]. PD can be sometimes viewed as “benign” in some tremor-dominant (TD) patients, as it progresses very slowly [9].

Some attempts were made to find specific biomarkers, which could help to differentiate or to link ET-PD patients to ET-only, or PD-only. All the studies dealing with this issue, published until 2011, were summarized in a thorough review of Fekete and Jankovic [15]. A few studies were published later, which add valuable information, and questions. Patients with ET–PD obtained significantly lower scores compared to ET-only on several cognitive tests, and showed more frequent familial history, and lower levodopa responsiveness [16]. ET patients who develop PD may have distinct pre-PD motor features than their counterparts with ET who do not develop co-existing PD [17]. Although the results of a follow-up radionuclide imaging study with ^123^I-Ioflupane (DaTscan) in ET patients did not support the hypothesis of a link between ET and PD, but in ET patients striatal binding was significantly reduced compared to healthy controls [18]. The most recent post-mortem study of 89 ET patients reported that 11 (12.4%) had pathological findings of progressive supranuclear palsy (PSP) [19]. This raised other questions, whether ET patients are at an increased risk of developing PSP, and what proportion of ET patients who develop presumed PD or Alzheimer’s disease, actually have PSP [19].

A hyperechogenicity of the substantia nigra (SN+) without any abnormalities in other brain structures is a common finding in transcranial ultrasound (TCS) of PD patients [20]. Recently, imaging by TCS has been recommended (European guidelines): 1) for the differential diagnosis of PD from atypical or secondary parkinsonian syndromes (PS); 2) for early PD diagnosis, and 3) for the detection of subjects at risk for PD [21]. However, TCS shows the SN + in a percentage of non-PD patients. This echo feature is found in up to 26% of ET patients, compared to approximately 10% of healthy controls [20,22,23].

The aim of the study was to look for a link or any distinguishing features by TCS. And to further elucidate the relationship between ET and PD, clinical examination findings in a group of patients who had already presented for a consultation with overlapping ET-PD phenotype were evaluated.

## Methods

### Design and subjects

A prospective observational case-control study was performed during a period from the 3^rd^ of January 2011, until the 30^th^ of January 2013, at the Hospital of Lithuanian University of Health Sciences (HLUHS, Kaunas, Lithuania). Initially, 433 subjects (332 patients and 101 controls) were referred for examinations from both Out-patient and In-patient units of the Neurology Department. Control subjects were recruited from the patients who were referred for carotid ultrasound and who had other neurological diseases, but not including movement disorders.

After one year all medical records of the patients were reviewed to see if the treating neurologists made any changes to the final clinical diagnoses. Only the ET-PD patients were invited for a re-consultation after a year, they were all contacted by phone. After the study, if a patient of any other group did not come for a re-consultation, the diagnosis was classified as recorded at the time of the last visit.

Of 332 patients directed for TCS, 46 patients (13.9%) were excluded from the study because of bilateral temporal acoustic bone insufficiency. In addition, 14 (4.9%) patients were excluded because their diagnoses changed or remained uncertain (N/A) after a follow-up: for 11 (2.5%) diagnosis remained N/A, for 1 (0.2%) diagnosis converted to atypical PS, and for 2 (0.5%) patients diagnosis changed to secondary PS.

Thus, 373 subjects were eligible for the final analysis: 272 patients and 101 controls. The patient group consisted of ET-PD (n = 15), PD-only (n = 141), and ET-only (n = 116). The higher percentage of all patients (68.3%) was referred for a *de novo* diagnosis. The majority of PD-only patients were at an early stage of the disease (80.1%). TD PD-only accounted for 48 (34%), non-TD–for 90 (63.8%), while 3 cases (2.1%) were N/A. When describing PD subtypes in more details: 48 (34%) patients were TD PD-only, 34 (24.1%)–postural instability gait disorder predominant (PIGD), 56 (39.7%)–mixed type, and the rest 3 (2.1%) already mentioned cases were N/A.

To serve the main purpose of the study, two important requirements were: 1) a full clinical characterization of the subset of patients with ET-PD phenotype; 2) an evaluation of TCS findings in order to determine the impact of this non-invasive, inexpensive, objective imaging tool in diagnostic and therapeutic decisions, when the latter phenotype is met in clinical practice.

### Ethics

Before the study was initiated, permission was obtained from Kaunas Regional Research Ethics Committee (No BE-2-70). All subjects recruited gave a written informed consent. Additionally, a written agreement from the patients was obtained to reproduce the images and video material anonymously.

### Clinical approach

During a visit of every subject, a neurological examination and a structured interview were performed (DŠ, BV, GD). The case history data included: duration of symptoms, topography, (a) symmetry, relation to voluntary movements, posture and task, heredity. The subjects were also asked about possible contact with toxic agents and history of head traumas. The most effective pharmacotherapy was recorded.

The diagnostic standard in our study was a clinical diagnosis, made by a treating neurologist. Inclusion criteria were: ≥18 years of age, a clinical diagnosis of idiopathic PD after follow up, which was based on widely accepted criteria: for probable PD–the United Kingdom Brain Bank criteria [24], for definite or classic ET–the consensus statement of the Movement Disorder Society [25], a clinical diagnosis with overlapping features of ET-PD (the Parkinsonian signs, such as bradykinesia, rest tremor, newly considered in previously diagnosed and well-documented ET-only patients), and TCS. Exclusion criteria were a bilateral temporal acoustic bone window insufficiency in the TCS examination or diagnosis of atypical, heredo-degenerative (cerebellar included), secondary PS, dystonia and unclear diagnosis, which didn’t meet the criteria for either ET or PD.

When clinically the diagnosis was debated, for those patients DaTscan (General Electric Healthcare, the United Kingdom) imaging was performed at the HLUHS, the Department of Nuclear Medicine. For the atypical cases, brain computed tomography (CT) and/or magnetic resonance imaging (MRI, Siemens Magnetom Avanto Syngo, 1.5 T) scans were made (RG).

For a thorough assessment, both objective and subjective questionnaires were applied. The clinician-rated scales that we used included the Unified Parkinson’s Disease Rating Scale (UPDRS), modified Hoehn and Yahr (H&Y) Stages Scale, and the Hospital Anxiety and Depression (HAD) Scale, the 36-item Short Form Survey (SF-36) that were self-rated [26-28].

### Ultrasonographic approach

TCS imaging was performed by one neurosonographer (KL). The sonographer was blinded to the clinical data, but still could see the examined subjects. We used a 2–5 MHz phased array transducer on a commercially available ultrasound system, Voluson 730 Expert (General Electrics Healthcare, Austria). With the subjects laid in a supine position, the transducer was placed over the pre-auricular temporal acoustic window bilaterally. The ultrasound beam was focused at a depth of 16.8/55Hz, manually adapting gain and degree of compression to allow optimal image quality at the mesencephalic, diencephalic and *sella media* planes. The SN + we classified by a plot of an area in cm^2^. The normative threshold values of the SN calculated in our laboratory were <0.20 cm^2^ (mean + 1 standard deviation, SD) and <0.26 cm^2^ (mean + 2 SD) [29]. The SN + was treated when it was enlarged at least on one side. For the ventricular system, the normative values were as follows: the third ventricle (V3) diameter <1.0 cm (mean + 2 SD); the lateral ventricles (LV) <2.1 cm (mean + 1 SD) (for the generation of the normative threshold values, see Additional file 1: Table S1).

### Statistical analyses

An analysis was performed with a statistical package SPSS version 19.0 (IBM, USA). Descriptive statistics were given as absolute numbers, percentages, mean ± SD, median and interquartile range (IQR). Normality of the variables was explored by the Shapiro-Wilk test. For the comparisons of non-parametric data, the Kruskal-Wallis test was used. For parametric data, the means between three or four groups were compared by analysis of variance (ANOVA), and between two groups, the Student t test was used. For categorical data χ^2^ criterion was used. Correlation analysis of non-parametrical data was performed by Spearman’s correlation, and of parametric, Pearson’s. A p value of less than 0.05 was used as the criterion for statistical significance.

## Results

### Clinical findings

The sample of the ET-PD patients accounted for 5.5% of all patients (15/272). The demographic and clinical characteristics of each patient of the ET-PD group are presented in Table 1. The mean age of ET-PD patients was 69 ± 9.6 years (yrs), 60% were female, median symptom duration was 6 yrs. In the ET-PD group, 11 of 15 (73.3%) patients had asymmetrical distribution of tremor at onset. The disease for the majority of patients in the PD-only group (n = 122, 86.5%) started asymmetrically, whereas for the majority (n = 81, 73%) in ET-only, it was bilateral (χ^2^ test, p < 0.001). Although, in the ET-only group some patients still did not fit into the category of classical ET-only, but exhibited the atypical features: 3 (2.6%) had cogwheel signs, 3 (2.6%) pyramidal signs, 2 (1.7%) postural instability and gait disorders, 2 (1.7%) subtle bradykinesia; and 31 (26.7%) had asymmetrical onset of tremor (χ^2^ = 233.5, p < 0.001). Out of 15 ET-PD patients, 8 (53.3%) had the onset of tremor after 50 yrs of age, and in 10 (66.7%) of them the ET-type tremor was present ≥5 yrs before the onset of PD-related symptoms. For 11 (73.3%) of ET-PD patients the mixed type (an action and/or posture plus of a rest) tremor-only was observed, and in 4 (26.7%)–the signs of bradykinesia/hypokinesia were detected, combined to an action and/or posture tremor. The topography of tremor in ET-PD patients on examination was as follows: arm(s)–in 15 (100%), head–in 9 (60%), leg(s)–in 3 (20%), voice–in 2 (13.3%). In PD-only patients tremor involved arm(s) in 133 (94.3%), leg(s) in 78 (55.3%), and head in 11 (7.8%) of cases (χ^2^ test, p < 0.001). A positive cogwheel sign was present in only 4 (26.7%) of ET-PD patients.

**Table 1:** The detailed demographic and clinical characteristics of every ET-PD patient

**No.**	**Symp. durat.**	**FH**	**Topography**	**Later.**	**Extr. tone**	**SN+**	**Pharmacotherapy**
1.	43	Yes	H + A	D	Yes	Yes	DA, amantadine 200 mg, propranolol 80 mg
2.	6	Yes	H + A	D	No	No	DA, clorazepate 10 mg
3.	21	No	A + L	B	No	Yes	Propranolol 120 mg, clonazepam 2 mg
4.	3.5	Yes	H + A	S	No	Yes	L-Dopa, pregabaline 75 mg
5.	10	No	H + A	D	No	Yes	DA, bromazepam 1.5 mg, vit. E
6.	30	Yes	H + A	D	No	No	L-Dopa
7.	6	No	H + V + A	D	Yes	Yes	L-Dopa, DA, propranolol 80 mg, citalopram,
							clonazepam 0.25 mg
8.	10	Yes	A + L	B	Yes	No	L-Dopa, propranolol 80 mg
9.	11	No	H + A + L	B	Yes	Yes	L-Dopa, DA, rasagiline 1 mg
10.	15	No	A	D	No	No	Sertraline, zolpidem
11.	3	No	A	S	No	Yes	Propranolol 40 mg
12.	45	Yes	A	S	No	Yes	L-Dopa, DA
13.	3	Yes	H + V + A	D	No	Yes	Clonazepam 2 mg, tiapride 150 mg
14.	1.5	Yes	H + A	S	No	Yes	Paroxetine, clonazepam 0.25 mg, vit. E, coenzyme Q10
15.	10	No	A	B	No	No	Gabapentine 100 mg, vinpocetine 20 mg, piracetam 400 mg, vit. E

More than a half (n = 8, 53.3%) of ET-PD patients denoted positive family history (FH) of tremor. Looking at the primary data (before an exclusion of the patients due to bilateral temporal acoustic bone insufficiency for TCS) in the ET-PD group (n = 17): none (0/17) of the patients from this group denoted positive family history of PD; 7/17 mentioned positive FH of ET type tremor among relatives: 6/17 tremor–mother (1 patient both mother and brother, and 1 patient both mother and father), 1/17 tremor-grandmother. Thus, in the PD group (n = 165): altogether 25/165 had positive FH: 9 had FH of possible PD (5/9 mother, 1/9 brother, 1/9 sister, 1/9 grandmother, 1/9 aunt); and 16 positive FH of ET type tremor (7/16 mother, 3/16 sister, 3/16 father, 1/16 aunt, 2/16- the data missing to whom of relatives exactly ET type tremor was positive).

Levodopa (n = 6, 40%), dopamine agonists (DA, n = 6, 40%), benzodiazepines (n = 6, 40%) and propranolol (n = 5, 33.3%) were prescribed separately or in combinations for the patients with ET-PD.

When comparing the ET-PD patient group to other groups of patients and controls (summarized in Table 2), statistically significant differences were found: between genders, there was a male (53.2%) predominance in PD-only, and a female (66.4%) in ET-only group (χ^2^ test, p = 0.007); within symptom duration, which was the longest (median 6 yrs) in the ET-PD group (Kruskal-Wallis test, p = 0.003); between the rate of positive family history, which was the most frequent (53.3%) in the ET-PD group (χ^2^ test, p = 0.005); in cogwheel sign, which was the most frequently detected (91.5%) in PD-only group (χ^2^ test, p < 0.001); in pyramidal signs, which were the most frequent (13%) both in ET-PD and PD-only groups (χ^2^ test, p = 0.009); also there were differences in the mean H&Y scores with the higher (2.6 ± 0.8) in ET-PD group (t test, p = 0.021). When comparing TD to non-TD PD-only groups, they did not differ in any clinical or demographic aspects, except for H&Y scores, which were higher in non-TD (1.96 ± 0.74 vs. 1.6 ± 0.70, t test, p = 0.009). As expected, the UPDRS part III points significantly differed between the PD-only vs. ET-only groups, with the highest scores being in the PD-only group (p = 0.021). We did not detect any significant differences in the results of the subjective clinimetric scales HAD and SF-36 subcomponents) between the patient groups, only to controls (Table 2).

**Table 2:** Demographic and clinical characteristics of ET-PD patients in comparison to ET-only, PD-only patients and controls

**Demographic and clinical characteristics**	**Patients (n = 272)**			***P *** value*	**Controls (n = 101)**	***P *** **value**^**#**^
	**ET-PD (n = 15)**	**ET-only (n = 116)**	**PD-only (n = 141)**			
Age, y	69 ± 9.6	63.9 ± 14.4	64.4 ± 11.2	NS	61.7 ± 12.4	NS
Gender (male)	6 (40)	39 (33.6)	75 (53.2)	0.007	56 (55.4)	0.004
Sympt. duration y	6 (3-15)	5 (1-10)	3 (1-6)	0.003^a^	N/A	N/A
Family history +	8 (53.3)	35 (31.3)	23 (17)	0.005	4 (4.3)	<0.001
Toxic exposure	3 (20)	19 (16.4)	23 (16.3)	NS	16 (15.8)	NS
Living in rural	4 (26.7)	19 (16.4)	29 (20.6)	NS	16 (15.8)	NS
Cogwheel sign	4 (26.7)	3 (2.6)	129 (91.5)	<0.001	0 (0)	<0.001
Pyramidal signs	2 (13.3)	3 (2.6)	19 (13.5)	0.009	10 (9.9)	0.025
H&Y stage	2.6 ± 0.8	N/A	1.8 ± 0.8	0.012	N/A	N/A
UPDRS part I	1 (0-1)	1 (0-3)	3 (1-4)	NS	N/A	N/A
UPDRS part II	5 (4-6)	13 (12-13)	11 (7-16)	NS	N/A	N/A
UPDRS part III	5 (3-6)	4 (1-6)	14 (9-20)	0.021^b^	N/A	N/A
HAD-Anxiety	1 (1-8)	6 (2-10)	8 (5-11)	NS	5 (3-8)	NS
HAD-Depression	4 (4-7)	4 (2-6)	6 (4-9)	NS	3 (1-7)	NS
SF36-PF	53.3 ± 17.6	52.3 ± 31.8	45.9 ± 25.3	NS	70 ± 24.2	0.001^c^
SF36-RLPF	16.7 ± 28.9	33.1 ± 29	33.3 ± 27	NS	50.2 ± 29.4	0.020^d^
SF36-RLEF	33.3 ± 57.7	51.4 ± 30	40.8 ± 34.2	NS	59 ± 32.2	NS^e^
SF36-EF	43.3 ± 10.4	43.8 ± 24.8	44.5 ± 19.4	NS	58.8 ± 17.1	0.007^f^
SF36-EW	66.7 ± 38.9	52.5 ± 26	53.7 ± 19.3	NS	64.7 ± 15.8	NS^g^
SF36-SF	58.3 ± 19.1	57.3 ± 31.5	53.8 ± 24.9	NS	75.7 ± 46.7	0.050^h^
SF36-P	66.7 ± 18.8	65.7 ± 26.7	49.5 ± 30.7	NS	61.7 ± 28.2	NS
SF36-GH	28.3 ± 2.9	35 ± 19.4	31.1 ± 16.9	NS	49.1 ± 18.5	<0.001^i^

Values are presented as mean ± SD or median with IQR, and numbers (percentages). *- p values were counted comparing 3 groups of patients, ^#^- p values were counted comparing 4 groups of subjects where available.

The post hoc multiple comparisons between the groups by Tukey HSD and LSD tests revealed significant differences:

^a-^ET-PD vs. PD-only p = 0.001, 95% CI 2.8-13.1, ET-only vs. PD-only p = 0.002, 95% CI 1.12-5.87; ^b^-LSD test only PD vs. ET-only p = 0.009, 95% CI 2.32-15.61; ET-PD vs. ET-only p = 0.008, 95% CI 4.86-31.08; ^c^-PD-only vs. control group p = 0.001, 95% CI 8.37-39.88; ^d^-PD-only vs. control group p = 0.05, 95% CI 0.01-33.83; ^e^-LSD test only, PD-only vs. control group p = 0.018, 95% CI 3.16-33.19; ^f^-PD-only vs. control group p = 0.011, 95% CI 2.48-26.16; ET-only vs. control group p = 0.024, 95% CI 1.46-28.67; ^g^-LSD test only, PD-only vs. controls p = 0.021, 95% CI 1.66-20.19; ET-only vs. controls p = 0.026, 95% CI 1.46-22.76; ^h^-PD-only vs. control group p = 0.039, 95% CI 0.77-43.1; ^i^-PD-only vs. controls p < 0.001, 95% CI 7.24-28.62; ET-only vs. controls p = 0.018, 95% CI 1.77-26.34; LSD test only, PD-only vs. ET-only p = 0.031, 95% CI 1.52-30.94.

A correlative analysis, taking into account all the groups, revealed significant correlations between: an age and HAD-Depression (r = 0.22, p = 0.034); the duration of symptoms and HAD-Anxiety (r = 0.26, p = 0.009), H&Y scale (r = 0.4, p < 0.001), UPDRS part I (Mentation, Behavior and Mood) scores (r = -0.33, p = 0.043).

### Ultrasonographic findings

The TCS findings in the patient groups and controls are given in Table 3. When comparing TD to non-TD PD-only patients, they differed in the mean largest SN (SN_Max_) plots, which were higher for non-TD group (0.35 ± 0.17 vs. 0.28 ± 0.25 cm^2^, t test, p = 0.015). Significant differences were detected: between the frequency of disrupted/absent nuclei raphe (χ^2^ test, p < 0.001), which were the most affected in ET-PD (38.5%) and PD-only (38.6%) groups; in hyperechogenic lentiform nucleus (LN+) rate, which was the most frequently (26.7%) observed in ET-PD group (χ^2^ test, p = 0.006); within red nucleus hyperechogenicity rate (χ^2^ test, p = 0.005), the most frequently visible in ET-only (17.2%) and the control (18.8%) groups (probably because the SN + did not overtake its area). Specifically in the subgroup of ET-PD patients, to whom ET-type tremor presented ≥5 yrs before the onset of PD-related symptoms (n = 10), the LN + was detected in only 2/10 (20%) of them (χ^2^ test, p = 0.088).

**Table 3:** Ultrasonographic (TCS) findings in the groups of patients and control subjects

**TCS parameters**	**Patients (n = 272)**			***P *** value*	**Controls (n = 101)**	***P *** **value**^**#**^
	**ET-PD (n = 15)**	**ET-only (n = 116)**	**PD-only (n = 141)**			
SN_R_ cm^2^	0.22 ± 0.12	0.20 ± 0.11	0.27 ± 0.14	<0.001^a^	0.15 ± 0.09	<0.001^d^
SN_L_ cm^2^	0.27 ± 0.15	0.23 ± 0.14	0.31 ± 0.16	0.001^b^	0.16 ± 0.09	<0.001^e^
SN_Max_ cm^2^	0.30 ± 0.16	0.24 ± 0.14	0.34 ± 0.16	<0.001^c^	0.18 ± 0.09	<0.001^f^
SN_Max_ ≥0.20 cm^2^	10 (66.7)	49 (42.2)	106 (75.2)	<0.001	18 (17.8)	<0.001
SN_Max_ ≥0.26 cm^2^	8 (53.3)	38 (32.8)	89 (63.1)		11 (10.9)	
V3 cm	0.69 ± 0.31	0.58 ± 0.28	0.58 ± 0.27	NS	0.53 ± 0.25	NS^g^
LV_R_ cm	1.81 ± 0.3	1.82 ± 0.26	1.78 ± 0.24	NS	1.79 ± 0.26	NS
LV_L_ cm	1.78 ± 0.26	1.81 ± 0.3	1.75 ± 0.24	NS	1.74 ± 0.26	NS
Raphe – or +/-	5 (38.5)	22 (20)	51 (38.6)	0.007	11 (13.1)	<0.001
Caudate +	0 (0)	2 (1.7)	2 (1.4)	NS	0 (0)	NS
Lentiform +	4 (26.7)	20 (17.2)	12 (8.5)	NS	4 (4)	0.006
Thalamus +	2 (13.3)	10 (8.6)	10 (7.1)	NS	10 (9.9)	NS
Red nucleus +	1 (6.7)	20 (17.2)	9 (6.4)	0.008	19 (18.8)	0.005

Values are presented as mean ± SD or numbers (percentages). *-p values were counted comparing 3 groups, ^#^-p values were counted comparing 4 groups.

The post hoc multiple comparisons by Tukey HSD and LSD tests showed statistically significant differences between the latter groups:

^a^-ET-only vs. PD-only p < 0.001, 95% CI 0.03-0.11; ^b^-ET-only vs. PD-only p < 0.001, 95% CI 0.03-0.12; ^c^-ET-only vs. PD-only p < 0.001, 95% CI 0.04-0.13; ^d^-ET-only vs. PD-only p < 0.001, 95% CI 0.03-0.11; ET-only vs. controls p = 0.032, 95% CI 0.003-0.09; PD-only vs. controls p < 0.001, 95% CI 0.07-0.16; ^e^-ET-only vs. PD-only p < 0.001, 95% CI 0.03-0.12; ET-only vs. controls p = 0.03, 95% CI 0.02-0.12; PD-only vs. controls p < 0.001, 95% CI 0.1-0.2; ^f^-ET-only vs. PD-only p < 0.001, 95% CI 0.04-0.13; ET-only vs. controls p = 0.003, 95% CI 0.02-0.12; PD-only vs. controls p < 0.001, 95% CI 0.1-0.2; by LSD only ET-PD vs. controls p = 0.019, 95% CI 0.02-0.17; ^g^-LSD test only ET-PD vs. controls p = 0.042, 95% CI 0.005-0.303.

DaTscan imaging was performed to 6 (40%) patients from the ET-PD group. For 3/6 (50%) patients a clearly reduced asymmetrical striatal radioligand binding was detected, i.e. signs for the degenerative Parkinsonism, for 2/6 (33.3%) - without evidence of dopaminergic dysfunction on DaTscan, and for 1/6 (16.7%) the patient-DaTscan result was debated, without a clear conclusion.

A correlative analysis, taking into account all the groups together, revealed a significant relationship between: the age and V3 diameter (r = 0.23, p < 0.001), the right (r = 0.18, p = 0.004) and also the left LV diameters (r = 0.25, p < 0.001); the SN_Max_ plot and V3 diameter (r = 0.15, p = 0.04).

## Discussion

The main echo findings of the present study in the patients with overlapping ET-PD phenotype, were similar to PD-only group, as more than two thirds (66.7%) of ET-PD patients had the SN + at a threshold value ≥0.20 cm^2^, and raphe nuclei interruptions or absence were detected in 38.5%. From the ultrasonographic comparative point, the closest comparison to our study was the recent investigation by Kim et al., focusing on TCS in 47 PD-only and 64 ET-only patients, in relation with putative pre-motor symptoms of PD [23]. The authors detected a significant association between SN + and each pre-motor symptom in the ET-only group, linking the results that SN + in patients with ET is influenced by the putative pre-motor symptoms of PD [23]. Combining the most prevalent non-motor features for PD to TCS imaging, including olfactory, autonomic (gastrointestinal) dysfunction, depression with anxiety and REM sleep behavioural disorder, would prove very useful [22,30].

**Figure 1:**
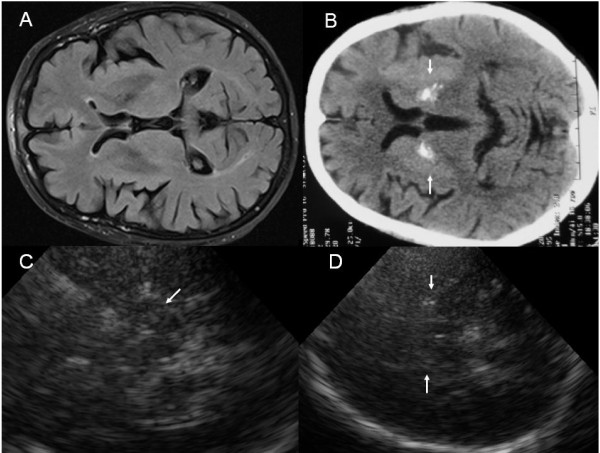
**Images of a patient with ET-PD-RLS, suggesting an alternative diagnosis of parkinsonism after paraclinical tests. A**-cranial MRI, T2W, **B**-cranial CT, markedly hyperintensive signals bilaterally in the lentiform nucleus (white arrows), **C**-TCS, mesencephalic plane, moderate SN and red nucleus hyperechogenicity (white arrow), **D**-TCS, diencephalic plane, bilaterally markedly hyperechogenic lentiform nuclei (white arrows).

The single distinguishing TCS feature, was an increased frequency (26.7%) of detected LN + in the group of patients with ET-PD (Figure 1), however significant when compared to controls only (χ^2^ test, p = 0.006). Usually such ultrasonographic findings are characteristic to PS [20,21]. In the study of Behnke et al., unilateral or bilateral LN + was found in 13/18 (72.2%) patients with PSP, in 23/32 (71.9%) patients with parkinsonian variant of multisystem atrophy (MSA-P), and in only 10 of 88 (11.4%) patients with idiopathic PD [31]. Taking into account the SN echogenicity and V3 diameter, the normal SN indicated MSA-P rather than PD, whereas V3 dilatation of >1.0 cm in combination with LN + indicated PSP rather than PD [32]. In the present study, when analyzing the subgroup of ET-PD patients, to whom ET-type tremor presented ≥5 yrs before the onset of PD-related symptoms, TCS helped to reveal the LN + in only 2/10 (20%) of them, this was indistinguishable even from the control group (p = 0.088). This is probably because a few patients, with a short disease duration (<5 yrs), who looked like they may be developing co-morbid PD clinically, may have had alternative diagnosis for PS (and were misclassified as having ET-PD). See an illustrative case (Figure 1) with clinical ET-PD plus restless legs syndrome (ET-PD-RLS), where different imaging methods were applied and the findings were described, suggesting an alternative diagnosis–idiopathic basal ganglia calcification (IBGC). Chronologically, first of all, cranial MRI was performed to this patient at another hospital, which was evaluated as normal, the patient was subsequently diagnosed as having ET-PD plus RLS at HLUHS and was then referred for TCS examination, followed by cranial CT scan, which confirmed TCS findings. These echo findings in ET-PD group have similarities to the results of the recent post-mortem study, where in 12.4% (11/89) of the ET patients atypical PS pathological findings were detected (ET-PSP) [19]. Also the frequency of pyramidal signs in our study, which were the most often detected (13%) both in ET-PD and PD-only groups (p = 0.009), might also indicate an alternative diagnosis in some cases (excluding those, to whom pyramidal signs can be explained by the central spinal stenosis with myelopathy, acute or residual cerebrovascular events and post-traumatic residual signs).

The significant differences between the patient groups were detected when comparing clinical results. In the ET-PD group male to female rate was closer to PD-only (40% and 53.2% respectively). There were similarities in the duration of symptoms between the ET-PD and ET-only groups (median 6 yrs and 5 yrs respectively). Chaudhuri et al. showed that longstanding isolated asymmetrical postural tremor may evolve to PD, and this was supported in 5 of the 13 reported cases (38%) by abnormal radionuclide imaging [33]. Whereas in 5 out of 24 cases, uncertain parkinsonian signs and normal imaging led to a change of the diagnosis to ET [33]. DaTscan imaging was performed to 6 (40%) patients from the ET-PD group, and for the majority (3/6, 50%) of patients a clearly reduced asymmetrical striatal radioligand binding was detected, i.e. signs for the degenerative Parkinsonism, thus for 2/6 (33.3%) - without clear evidence of dopaminergic dysfunction on DaTscan. Ceravolo et al emphasized that the clinical presentation of isolated tremors (of unilateral postural, unilateral mixed or unilateral rest) is insufficient to allow a precise early-stage diagnosis, as PD tremor could be very different ranging from typical rest to unilateral postural or mixed tremor, whereas detection of presynaptic dopaminergic dysfunction by SPECT could lead to diagnosis of atypical tremor disorders at very early stage [34].

According to the UPDRS part III points, the significant differences were detected between PD-only vs. ET-only groups as expected, with the highest scores, in PD-only group (p = 0.021). There were no significant differences between ET-PD vs. PD-only groups, with just a tendency of the less severe Parkinsonism in ET-PD patients than that of PD patients (median 5 vs. 14 points). The similar findings were detected in a retrospective study by Simões and colleagues [17].

A positive family history rate was highest in the ET-PD group. This feature was similar to ET-only group (53.3% and 31.3% respectively). Even though ET is one of the most common genetic disorders, it is yet to be identified as being a specific gene mutation. Puschmann et al. extensively investigated large kindred whose family members had PD, ET, RLS and depression, an overlap (mainly ET-PD or PD-RLS) was detected in 64% (7/11) of these individuals [35]. The fact that the risk of ET is significantly increased in relatives of patients with PD, suggests the possibility that both conditions are genetically related and probably share common hereditary predisposition [6]. In the recent study of Barut et al., the patients with ET–PD displayed a higher frequency of familial tremor history, as was recorded in our study [16]. The same authors also found significantly lower scores compared to the ET-only group on several cognitive tests, and lower levodopa responsiveness [9,16].

Our sample of the ET-PD patients, which accounted for only 5.5% of all patients, was small (rare phenotype) and quite heterogeneous itself. The small number of cases with this phenotype was also reported in other studies: n = 9 [16], and n = 18 out of 387 cases with Parkinson and 110 with tremor [17]. This causes speculation to support the hypothesis that the linkage between the two separate disorders could be rather coincidental [8]. However, to clearly support or deny the hypothesis about the higher risk for the patients with ET-only to develop PD, another study design (a larger cohort, follow-up) is necessary.

For most of the ET-PD patients the anti-parkinsonian medications that were prescribed (60%), less than a half (40%) responded well to levodopa, and the majority (66.7%) required additional medications traditionally used for ET.

We noticed a dissociation between the presence of a positive cogwheel rigidity in 26.7% of the ET-PD patients vs. 91.5% in the PD-only group, but a much higher mean of H&Y scores (2.6 ± 0.8 vs.1.8 ± 0.8 respectively). Moreover, in the majority of the ET-PD group, 11/15 (73.3%) of patients had asymmetrical distribution of tremor, and 8/15 (53.3%) had an onset of tremor after 50 yrs. The results of the radionuclide imaging study by Coria et al. suggested that the current diagnostic criteria for ET should be revised to include asymmetry and late-onset tremor as predictors of nigrostriatal denervation [10]. This is because often found isolated action tremor is a frequent presenting symptom in a subset of individuals with PD, who are often misdiagnosed as having ET [10,34].

The main limitations of our study were that there was no pathological or functional imaging confirmation for all ET-PD patients. Also there was a limited time for a follow-up.

## Conclusions

The main TCS findings of the present study in patients with overlapping ET-PD phenotype were similar to PD-only group, taking into account the rate of the SN + and of the red nucleus, the raphe nuclei disruptions or absence. The clinical features linking ET-PD to PD-only group were an asymmetrical and late-onset tremor for the majority of ET-PD patients, and the presence of a rest tremor on examination. Some ET patients, who look like they may be developing co-morbid PD clinically, may have alternative diagnosis for Parkinsonism, which can be delineated by TCS examination. The highest positive family history rate among ET-PD patients, compared to ET-only and PD-only groups, indicates strong hereditary predisposition and needs genetic underpinnings. An addition of longitudinal follow-up studies is substantially needed to increase diagnostic certainty, also the confirmatory neuropathological studies of ET-PD clinical phenotype are required to describe and quantify the pathological changes.

## Abbreviations

ET: Essential tremor; PD: Parkinson’s disease; ET-PD: Overlapping phenotype with both ET and PD features; PS: Parkinsonian syndrome; PSP: Progressive supranuclear palsy; MSA-P: Multisystem atrophy, parkinsonian variant; RLS: Restless legs syndrome; CT: Computed tomography; MRI: Magnetic resonance imaging; DaTscan: Single photon computed tomography with ^123^I-Ioflupane; TCS: Transcranial sonography; SN: Substantia nigra; LN: Lentiform nucleus; NR: Raphe nuclei; VL: Lateral ventricle; V3: Third ventricle; TD: Tremor dominant; H&Y: Hoehn and Yahr stage scale; UPDRS: The Unified Parkinson’s disease Rating Scale; HAD: Hospital Anxiety and Depression Rating Scale; SF36: 36-item the Quality of Life Questionnaire, short form; PF: Physical function; RLPF: Role limitations physical functioning; EF: Emotional function; RLEF: Role limitations emotional functioning; SF: Social functioning; EW: Emotional vitality; P: Bodily pain; GH: General health; KL: Kristina Laučkaitė; DR: Daiva Rastenytė; DŠ: Danguolė Šurkienė; BV: Birutė Vaidelytė; GD: Gabrielė Dambrauskaitė; AS: Andrius Sakalauskas; AV: Antanas Vaitkus; RG: Rymantė Gleiznienė; SD: Standard deviation, 95% CI- 95% confidence interval; IQR: Interquartile range; yrs: Years; HLUHS: The Hospital of Lithuanian University of Health Sciences; NS: Not significant; N/A: Not applicable; FH: Family history; H: Head; A: Arm; V: Voice; L: Leg; D: Right; S: Left; B: Bilateral; Symp. durat: Symptom duration; Lat.: Lateralization; Extr.: Extrapyramidal; L-Dopa: Levodopa; DA: Dopamine receptor agonists; + hyperechogenic: - or +/- absent or disrupted; Max.: Biggest; FH: Family history.

## Competing interests

KL received speaker honoraria from GlaxoSmithKline for giving a lecture about diagnostics of Parkinson’s disease. BV received a research grant from the Research Council of Lithuania for an analysis of the non-motor symptoms in parkinsonian disorders (No SMT12P-163). Other authors declare no competing interests.

## Authors’ contributions

KL made ultrasound evaluation, conceived of the study, performed the statistical analysis, drafted the manuscript. BV, GD and DŠ performed clinical evaluation of the patients and controls, applied the scales and interviews. RG performed brain MRI and CT imaging. AS helped with preparing the draft of the manuscript. AV participated in the design of the study. DR participated in its design and coordination, also revised the manuscript critically. All authors read and approved the final manuscript.

## Pre-publication history

The pre-publication history for this paper can be accessed here:

http://www.biomedcentral.com/1471-2377/14/54/prepub

## Supplementary Material

Additional file 1: Table S1 Normative reference TCS values at the Hospital of Lithuanian University of Health Sciences. Click here for file
