# Supplementary material for: Ultrasonographic (TCS) and clinical findings in overlapping phenotype of essential tremor and Parkinson’s disease (ET-PD)
Source: BMC Neurol. 2014 Mar 22;14:54. doi: 10.1186/1471-2377-14-54 (PMC3998107; doi:10.1186/1471-2377-14-54)
Supplement: Additional file 1: Table S1 — Normative reference TCS values at the Hospital of Lithuanian University of Health Sciences. [file 1471-2377-14-54-S1.docx]

**Additional file**

**Table S1 - Normative reference TCS values at the Hospital of Lithuanian University of Health Sciences**

| Age terciles  (n=100) | SN_R_  (mean±  SD, cm^2^) | SN_L_ (mean±  SD, cm^2^) | SN_Max_  (mean±  SD, cm^2^) | V3  (mean±  SD, cm) | VL_R_ (mean±  SD, cm) | VL_L_ (mean±  SD, cm) |
| --- | --- | --- | --- | --- | --- | --- |
| ≤54 y | 0.09±0.06 | 0.10±0.05 | 0.12±0.03 | 0.24±0.14 | 1.61±0.19 | 1.53±1.78 |
| 55-73 y | 0.14±0.06 | 0.13±0.06 | 0.16±0.06 | 0.55±0.22 | 1.81±0.19 | 1.75±1.91 |
| ≥74 y | 0.08±0.05 | 0.16±0.09 | 0.17±0.07 | 0.81±0.17 | 2.16±1.99 | 2.1±0.15 |
| Total | 0.11±0.07 | 0.12±0.06 | 0.14±0.06 | 0.46±0.27 | 1.76±0.25 | 1.68±0.24 |
| Generation of the normative TCS values: | | | | | | |
| +1 SD | 0.18 | 0.18 | **0.20** | **0.73** | **2.01** | **1.92** |
| +2 SD | 0.25 | 0.24 | **0.26** | **1** | **2.26** | **2.16** |
| *P value** | 0.506 | 0.218 | 0.044 | 0.080 | 0.857 | 0.898 |

The values were counted by performing TCS examination for 100 healthy controls with ultrasound machine Voluson730 Expert (GE Healthcare, Austria) with a commercially available 2-5 phased array (PA) transducer. The values are presented as a mean ± standard deviation. *A *P* value was counted when comparing the three groups of subjects according to age terciles.

Abbreviations: SN- the substantia nigra, R- right, L- left, Max- the biggest plot, y- year, SD- standard deviation, V3- the third ventricle, VL- lateral ventricle.
